# Supplementary material for: Content-rich biological network constructed by mining PubMed abstracts
Source: BMC Bioinformatics. 2004 Oct 8;5:147. doi: 10.1186/1471-2105-5-147 (PMC528731; doi:10.1186/1471-2105-5-147)
Supplement: Additional File 5 — The original Chilibot query results of the term "long-term potentiation (LTP)" and 22 other terms, limiting the latest references analyzed to the years 1990, 1995, 2000, and 2004. [file 1471-2105-5-147-S5.bz2 › chilibotAdditionalFile5/ltp1995/html/PKA_AMPA.html]

 


 **PKA** and **AMPA** 
  
Found 7 abstracts in PubMed,  **7 abstracts were retrieved and analyzed**.  


---

 Search Google  |
 PDF files only 
|  EDU domain only 

---

**Interactive relationship** (e.g. stimulation, inhibition, etc)

- Furthermore, depression of these currents by a competitive inhibitor of  **PKA**  indicates that  **AMPA**  kainate receptors are regulated by endogenous  **PKA** .  Ref: 1653455 Science, 1991
- Forskolin treatment gave a three to fourfold increase in the active catalytic subunit of  **PKA**  but did not result in the 32P labeling of  **AMPA**  type GluRs, CaM kinase II, or MARCKS.  Ref: 7509863 J Neurosci, 1994
- Currents induced by activation of the  **AMPA**  kainate receptor were potentiated by agents that specifically stimulate adenosine monophosphate cAMP dependent protein kinase A  **PKA**  activity or were supported by intracellular application of the catalytic subunit of  **PKA**  by itself or in combination with cAMP.  Ref: 1653455 Science, 1991

- :-)
